# Can mixed assessment methods make biology classes more equitable?

**DOI:** 10.1371/journal.pone.0189610

**Published:** 2017-12-27

**Authors:** Sehoya Cotner, Cissy J. Ballen

**Affiliations:** Department of Biology Teaching and Learning, University of Minnesota, Minneapolis, Minnesota, United States of America; University of Westminster, UNITED KINGDOM

## Abstract

Many factors have been proposed to explain the attrition of women in science, technology, engineering and math fields, among them the lower performance of women in introductory courses resulting from deficits in incoming preparation. We focus on the impact of mixed methods of assessment, which minimizes the impact of high-stakes exams and rewards other methods of assessment such as group participation, low-stakes quizzes and assignments, and in-class activities. We hypothesized that these mixed methods would benefit individuals who otherwise underperform on high-stakes tests. Here, we analyze gender-based performance trends in nine large (*N* > 1000 students) introductory biology courses in fall 2016. Females underperformed on exams compared to their male counterparts, a difference that does not exist with other methods of assessment that compose course grade. Further, we analyzed three case studies of courses that transitioned their grading schemes to either de-emphasize or emphasize exams as a proportion of total course grade. We demonstrate that the shift away from an exam emphasis consequently benefits female students, thereby closing gaps in overall performance. Further, the exam performance gap *itself* is reduced when the exams contribute less to overall course grade. We discuss testable predictions that follow from our hypothesis, and advocate for the use of mixed methods of assessments (possibly as part of an overall shift to active learning techniques). We conclude by challenging the student deficit model, and suggest a course deficit model as explanatory of these performance gaps, whereby the microclimate of the classroom can either raise or lower barriers to success for underrepresented groups in STEM.

## Background

Women who enter college in any of the STEM (science, technology, engineering and mathematics) disciplines exhibit greater attrition than do their male peers, a gap that continues throughout most STEM professions [1]. Previous research has offered many explanations for the gender gap in STEM retention and performance [e.g., in biology [2], physics [3,4], engineering, chemistry [5], and math [6]]. Some explanations for this phenomenon relate to student preparation or academic abilities, which is collectively known as the *student deficit model* [7]. This model focuses on inadequacies of individuals, assuming some students enter college lacking the academic resources necessary to succeed in an otherwise fair learning environment. According to this model, high achievement is the direct result of hard work and inherent abilities.

Other explanations for the gender gap include the negative effect of environmental conditions on student performance [8–13], which we define here as the *course deficit model*. In this model, classroom practices favor certain groups of students while increasing performance disparities. In order to clarify the significance of student achievement in response to different classroom practices, we can manipulate the classroom environment and measure the outcomes (e.g. performance, retention, attitudes) for students.

For example, previous work has demonstrated that within the classroom, gender-biased gaps in performance may be explained in full or in part by female susceptibility to *stereotype threat* (ST; [6,14], whereby in high-stakes testing situations individuals conform to a perceived stereotype—in this example, the stereotype that women have less capacity to succeed in STEM [15]. Key criteria for ST are an awareness of a negative stereotype, and some type of stress- or risk-inducing scenario such as a high-stakes test or job interview [16]. According to the ST hypothesis, individuals under threat, subconsciously aware of stereotypes about their competence in these disciplines or skills, may have mental energy and focus diverted from the content or skill itself [17]. The ST phenomenon has been demonstrated in many disciplines, with special emphasis on math disparities [18,19].

A recent examination of 116 courses at the University of Michigan [5] found gendered performance differences on high-stakes exams such as midterms and finals—a phenomenon that is, critically, most pronounced in STEM courses. Because these differences were largely absent from non-STEM courses, the investigators concluded that ST may be at work, because of prevailing stereotypes about female [in]competence in STEM. If differential performance is a result of ST, then we would expect women to underperform—relative to their male counterparts—on high-stakes exams, but not on lower-stakes course assessments (homework, lab reports, small-value quizzes, etc.), presumably because the lower-stakes assessments pose less risk to the student. In fact, in recent work [20], we demonstrated that in women, but not in men, test anxiety negatively predicts exam performance, a finding that transcends students’ level of preparation.

For the current study, we hypothesized that the use of mixed assessment methods, instead of complete reliance on high-stakes exams, disproportionately benefits women in introductory biology. Women may respond negatively to the risk associated with high-stakes tests [20,21], but do not perceive high risk during other forms of assessment. Specifically, we predicted that females would underperform on high-stakes exams, but not on the portion of the grade due to lower-stakes assessments (e.g., lab work, written assignments, weekly quizzes, etc.). Further, we predicted that as grading schemes shifted to de-emphasize exams, overall performance gaps between males and females would be minimized or eliminated.

To test these predictions, we first evaluated student performance in nine high-enrollment introductory biology courses at a large public university. Specifically, we analyzed exam scores, total course grade, and combined performance on various lower-stakes assessments—all as a function of gender and incoming preparation (e.g., comprehensive American College Test, or ACT, scores). Second, we conducted three ‘case study’ analyses of courses that shifted grading schemes to either emphasize or deemphasize the influence of exam performance on final course grades. Specifically, we analyzed four different courses—one in which the grading scheme changed to place greater emphasis on exam scores, one in which the grading scheme changed to de-emphasize exam scores, and a pair of sequential courses with different grading regimes, but identical student populations.

## Methods

### Student populations

For our first study, we investigated predictors of course performance in nine large introductory courses taken in fall 2016 by non-biology majors (Table 1). Courses ranged in size from 90 to 239 students, and varied in the proportion of the total course grade that was due to exams (midterms and finals). In one course, for example, 41% of the course grade was calculated from exam scores, but in another, 52% of the grade was due to exams. Specifically, we categorized grades as *exam grade* (combined performance on midterms and finals), *non-exam grade* (performance on any non-exam assessments), and *final course grade* (a combination of *exam grade* and *non-exam grade*). A third-party individual, not involved in the course or this research, matched student grades to student gender, age, and incoming academic preparation (American College Test, hereafter ACT).

**Table 1:** Characteristics of nine introductory-biology courses analyzed for performance disparities.

Class section	Instructor	Class N	Percentage (%) exam in total course grade	Average Age	Average ACT per class section (SD)
1	A	115	41	20.83	25.75 (3.42)
2	A	115	41	20.70	26.46 (3.55)
3	B	182	41	20.38	26.62 (2.81)
4	C	95	46	20.18	26.98 (3.81)
5	C	90	46	19.68	28.05 (3.17)
6	D	229	50	20.04	26.89 (3.80)
7	E,F	153	52	20.29	26.91 (3.55)
8	E,F	178	52	20.06	26.32 (3.53)
9	G	239	52	20.18	26.08 (3.55)

For the second study, we focused on three different case studies in which grading schemes were changed (summarized in Table 2):

1. In Biology 100 (introductory biology for nonmajors), study populations included students enrolled in two separate semesters of the course, one (in Spring 2016) in which exams contributed 28% to total course grade, and one (in Spring 2017) in which exams contributed 44% to total grade. All exams are multiple-choice questions only, and machine-graded. Other than the grading scheme, all other aspects of the course were similar between the two semesters, including the instructor, topics covered, and lab activities.
2. In Biology 300 (an upper-level Evolution course), populations included students enrolled in two separate semesters of the course, one (in Spring 2016) in which exams contributed 50% to total grade, the other (Fall 2016) in which exams contributed 30% to final grade. Exams are short-answer format, and graded by teaching assistants using an instructor-developed rubric. Again, other than the grading scheme, all other aspects of the course were similar between the two semesters, including the instructors, topics covered, and lab activities.
3. The third case involved a two-semester sequence of courses (introductory biology for biology majors), in which the instructor and student populations were nearly identical, but the grading schemes varied. In the first semester of the sequence, Biology 202, exams contributed 22% to total grade, but in the second semester, Biology 203, exams contributed 42% to total grade in the course. Exams include both multiple-choice and short-answer items. Multiple-choice items are machine-graded, while short-answer items are graded by professors and teaching assistants using an instructor-developed rubric. In this analysis, we only examine students who took *both* Biology 202 and Biology 203.

**Table 2:** We compared three courses that changed grading schemes over the course of two semesters.

Course	Academic level	Major or nonmajors	Instructor identification	Student cohorts	Semester 1 (% exam)	Semester 2 (% exam)	Direction of exam % over time
BIOL 100	1^st^ year	Nonmajors	Same both semesters	Different	Sp2016 (28)	Sp2017 (44)	Increase
BIOL 202/203	2^nd^ year	Majors	Same both semesters	Same	Sp2016 (22)	Fa2016 (42)	Increase
BIOL 300	3^rd^ year	Majors	Same both semesters	Different	Sp2016 (50)	Fa2016 (30)	Decrease

### Statistical analysis

We performed all statistical analyses using SPSS software version 24 (SPSS Inc., Chicago, IL, USA). We used multilevel modeling with hierarchically nested data (students in different classes) to account for the non-independence of data in nested-data structures [22,23]For analyses we used the Akaike’s information criterion (AIC) to assess model significance [24]. AIC allows us to estimate the best model for our data, based on an estimation using AIC differences (Δ*i =* AIC_model_ *i*–minAIC, where minAIC is the model with the smallest AIC value). We performed four separate sets of analyses. We were interested in the interaction between the percentage that exams contribute to the final course grade (PercExam, a continuous fixed effect) and student gender (SGender, a fixed effect with two levels). Therefore, our model initially included those three effects (SGender, PercExam, and SGender*PercExam) and ACT score. We included ACT score to account for variation in students’ incoming preparation for the courses [25]. In addition, we tested whether the following variables improved the fit of the model for the given set of data using AIC differences: (1) student underrepresented minority status (whether they are African American, Hispanic, Native American, or Pacific Islander; hereafter URM, a factor with two levels); (2) student age (Age); (3) class size. Only students with a complete set of these variables were included in these analyses. We ultimately chose the most parsimonious model that best fit the data. The final model for exam performance, non-exam performance, and total course performance included the following predictor variables:

PerformanceMetric=ACTcompositescore+SGender+Age+PercExam+SGender*PercExam+(ClassSec)

Class section (ClassSec) was included as a random effect, and was tested for significance by removing it and taking the difference between the -2 log likelihoods. This was tested against a chi-square distribution with one degree of freedom.

Next, we conducted three ‘case study’ analyses of courses that shifted grading schemes to either emphasize or deemphasize the influence of exam performance on final course grades (Table 2). For the first two cases, we used univariate general linear models to compare metrics of student achievement across two semesters of BIOL 100 and 300. With average exam grade and total course score as the dependent variables, we included ACT score, SGender, semester, and the interaction between SGender and semester for each analysis. An ANOVA showed that incoming ACT scores did not differ significantly between semesters (for women in BIOL 100 *F*_1,17_ = 0.932 *P* = 0.539, for men in BIOL 100 *F*_1,14_ = 0.481 *P* = 0.935; for women in 300 *F*_1,15_ = 0.532 *P* = 0.913, for men in 300 *F*_1,14_ = 0.802 *P* = 0.663), indicating that incoming student populations were comparable in their preparation. We did not have ACT scores for seven students in BIOL 100 and nine students in 300, and so we assigned average ACT scores for their classes to those students in order to include them in the analyses. Further sensitivity analyses, in which we tested one standard deviation increase and decrease (±SD) of the ACT input for those students, did not significantly change our results.

For the third case study, we focused on a two-semester sequence of courses restricted to lower-division majors in biology. BIOL 202 and BIOL 203 are two courses taken consecutively by students, and so a high proportion of students who took BIOL 202 in the spring of 2016 also took BIOL 203 the following fall (97% of students in BIOL 203 took BIOL 202 the previous semester). In these courses, we were interested in individual students’ performance in the two classes, which are similar in nature (‘Part 1’ and ‘Part 2’ of a Foundations of Biology for Biological Science Majors sequence) but differ in the extent to which exams make up the final grade. To analyze these courses, we used a mixed model, wherein we included student ID as a repeated measure across semesters, and used a first-order autoregressive (AR1) covariance matrix. With this covariance matrix, we assume that residual errors within each subject are correlated, but are independent across subjects. With average exam grade and total course score as the dependent variables, we included ACT score, SGender, semester, and the interaction between SGender and semester for each analysis. We used Pearson correlations to examine whether baseline estimates (data collected prior to the course) were correlated with each other and with student outcomes. We deleted one outlier found in the residuals in our analysis of students’ exams in order to meet the assumptions of a mixed model. This individual had an average exam score of 20% across the semester (whereas the next lowest cumulative score for students was >60%). For all ‘case study’ analyses, we report post-hoc Bonferroni pairwise comparisons to clarify performance outcomes of students based on gender.

## Results

### Do high stakes exams drive gender gaps across nine introductory biology classes?

**Fig 1:**
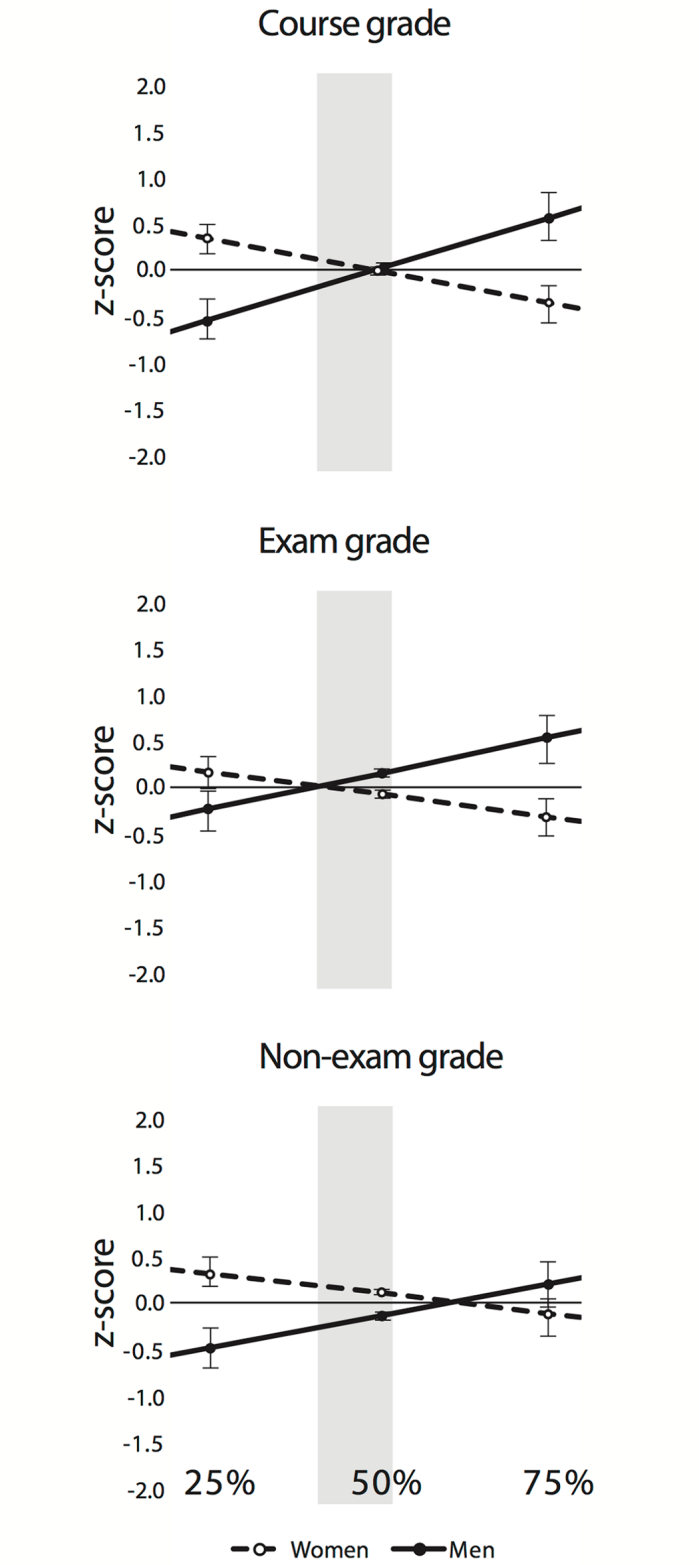
Expected and observed standardized course grade, exam grade, and non-exam grade (±SE) for women (dashed line, triangles) and men (solid line, squares) in biology when the exam accounts for 25%, 50%, or 75% of the final grade. Expected values are derived from estimated marginal means of performance outcomes. All values are represented as z-scores, which are negative when the students’ raw scores are below the mean, and positive when above. Solid and dashed lines represent expected performance based on student grades from nine introductory biology courses at the University of Minnesota. The shaded region of the graph represents courses for which we have data.

In nine introductory biology courses for nonmajors (*N* = 1078), women underperformed on exams compared to men as the percentage of exams contributing to total course grade increased (*B* = − 2.205, *t*(1063) = -2.032, *P* = 0.042, *SE* = 1.085), and this was also true for their course performance overall (*B* = − 2.983, *t*(1071) = -2.601, *P* = 0.009, *SE* = 1.147). However, we found no such gender differences in non-exam grades (*B* = -1.574, *t*(1071) = -1.258, *P* = 0.209, *SE* = 1.252; Tables A-C in S1 File; Descriptive statistics in Tables D-E in S1 File).We used the statistical models to display estimated marginal means of the performance outcomes. This is a testable predictive model which suggests that as exam value increases, the performance gaps between men and women will increase (Fig 1), and as exam value decreases, performance gaps will also emerge, with women outperforming men.

Next, we explored whether women are underperforming because of the effect of larger class sizes, which generally rely more on exams to assess students because it is logistically difficult to emphasize non-exam grades for many students. To investigate this, we examined whether class size and the percentage that exams account for in the final grade are correlated. We did not find a significant correlation between these two continuous variables (Pearson correlation = 0.535; *P* = 0.137). A second explanation may be that a gender gap exists in some courses and not others. We did not have the luxury of designing courses in fall 2016 such that some sections had high-stakes exams and others low-stakes exams, which would have allowed us to compare high-stakes sections to low-stakes sections of identical courses. In order to compare performance within a single course, we identified three biology courses that shifted grading schemes to either emphasize or de-emphasize exam performance as it contributes to final course grades.

### Three case studies in biology: How do students perform after significant changes in class grading scheme?

In BIOL 100 (*N* = 230), women’s performance on exams relative to men’s *decreased* significantly as the instructor shifted the percentage of exams contributing to total course grade from 28% in spring 2016 up to 44% in spring 2017 (Semester*SGender interaction *F*_1,3.6_ = 4.29, *P* = 0.04, Table F in S1 File). In contrast, women’s performance on exams relative to men’s *increased* significantly when instructors in 300 (*N* = 164) shifted the percentage of exams contributing to total course score from 50% in spring 2016 down to 30% in fall 2016 (*F*_1,2.8_ = 4.75, *P* = 0.03; Table G in S1 File).

In BIOL 202/203, a two-semester sequence, instructors shifted the percentage of exams contributing to total course grade from 22% in spring 2016 up to 42% in fall 2016. In this course, we only follow performance of students who took both courses in this sequence (*N* = 155). Again, women’s performance on exams relative to men’s *decreased* significantly as the instructor increased the percentage that exams contributed to total course grade (*F*_1,155_ = 4.37, *P* = 0.04; Table H in S1 File).

**Fig 2:**
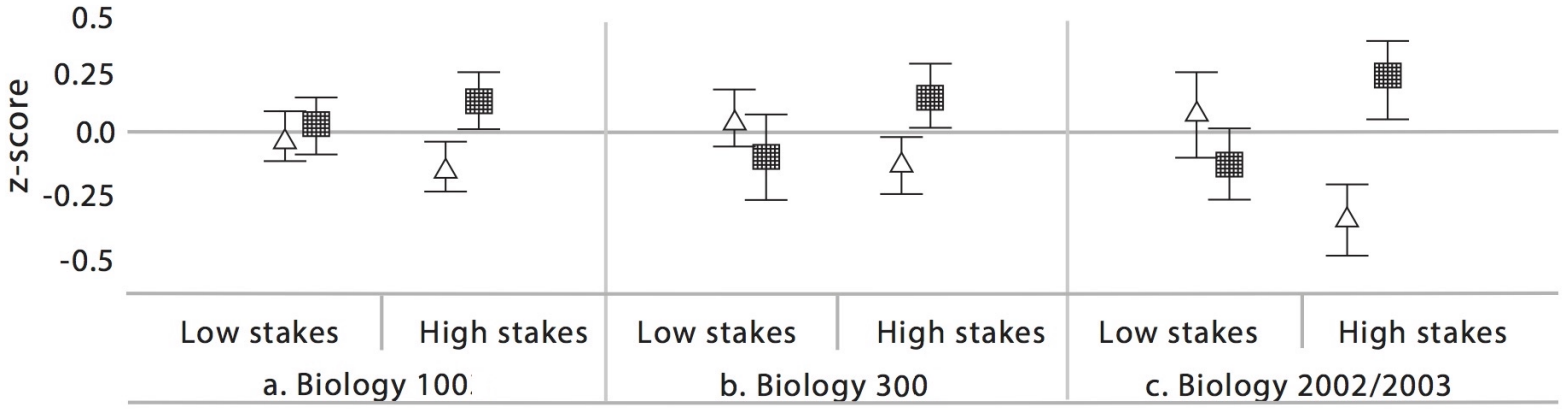
Gender-based performance on combined exam scores for three case studies: a. Biology 100; b. 300; and c. the Biology 202/203 sequence. Individual Z-scores for each exam are averaged and combined into total exam performance for women (triangles) and men (squares) across two semesters—for each course, one semester in which exams contributed over 40% of the course grade (“high stakes”) and one semester in which exams contributed less than 40% of the course grade (“low stakes”). There was a significant gender x semester interaction effect for performance in all three cases (p<0.05; Tables F-H in S1 File).

Fig 2 illustrates gender-based performance on exams during relative high-stakes and low-stakes semesters, for each of the three courses discussed above. We find support for the idea that, as exam value increases, gender-based performance gaps increase. The converse—that as exam value is minimized, gaps increase to favor women—is not supported by our case-study findings. All de-identified data are available in S1 Table.

## Discussion

### The role of mixed assessment methods in reducing performance gaps

We find that, across several introductory biology courses, women underperform on high-stakes exams relative to their male counterparts, a gap that does not exist on other, lower-stakes assessments. And, to the extent that exam scores contribute to overall course grade, these performance gaps carry over to final grade in a course. That is, as exams account for more of the total course grade, performance gaps are greater. When exams account for less of the total grade, gaps are minimized or eliminated entirely.

These gender gaps in performance are not exclusively mathematical—that is, it is not simply reducing the impact of exams that reduces gaps in total scores. Rather, our results suggest that *exam performance itself* is affected by the potential impact of the exam. When exams are worth less in the course, females perform on the exams as though they perceive less risk. The combined effect—underperformance on higher-stakes tests themselves, plus the cumulative impact of these tests on a student’s overall grade—results in female underperformance in biology courses.

To isolate the impact of grading schemes from other variables (different instructors, different student populations, variable in-class teaching techniques), we investigated three cases in which grading schemes changed over time. We consistently observed significant gender gaps in student performance during ‘high stakes’ semesters, with women underperforming compared to men. These gaps are absent in the comparative ‘low stakes’ semester.

### Is stereotype threat at work?

Females appear to respond to high-stakes exams as a higher risk, and underperform as would be expected if they were under stereotype threat in these biology courses. However, we did not establish—via surveys, interviews, or any sort of contextual manipulation—the salience of a stereotype about female deficiencies in biology. Thus, we are reluctant to make any claims about stereotype threat affecting females in these introductory biology courses, a finding aligned with that of others [26].

### Are the benefits of active learning due to mixed assessment methods?

Many investigators have documented a reduction in achievement gaps in active-learning environments [27,28] relative to traditional classroom environments. In these discussions, “active learning” refers to classrooms in which students are engaged in constructing their own knowledge [29]. Active learning techniques vary, and can include group work, case studies, modeling exercises, and a diversity of in-class assessment techniques.

The evidence for performance gains in active-learning environments is compelling [28–30] and broad. However, a significant gap in the literature is the mechanism by which these improvements occur. By incentivizing students to participate through mixed methods of assessment (a key feature of active learning), we reward consistent, ongoing preparation rather than performance on a few high-stakes examinations. In fact, modifying the value of exams in order to lower risk improves female performance on these exams, underscoring the fact that for some individuals, performance on exams may not reflect a student’s actual content knowledge [31,32]. Importantly, the lower-value exams assessed the same content knowledge as the high-value exams, a finding that should assuage concerns that low-stakes testing means a watering-down of expectations.

We propose, based on our current findings, that much of the performance gains documented by active learning may be due to the use of mixed assessment methods, whereby instructors use a diverse combination of formative and summative assessment techniques—both low- and high-stakes. This revised hypothesis—that *mixed assessment methods are a mechanism by which the transformation to active learning disproportionately benefits underrepresented groups in STEM*—is supported by the data we present here. However, we envision many testable predictions that follow. First, mixed assessment methods should be a signature of classes that have been transformed to highlight active learning. Also, if mixed assessment methods are a key feature of gains associated with active learning, then instructors that migrate to teaching with multiple low-stakes assessments should see patterns in performance similar to those we describe above. Finally, if risk perception is culpable in the underperformance of women on high-stakes exams, efforts to minimize perceived risk should reduce the gender differences we [20]—and others—have documented for exams in STEM disciplines. We urge our colleagues to join us in testing these predictions, investigating their own courses and mining local, institutional data using techniques described above.

If our predictions are confirmed, the actionable items are simple and straightforward to implement: instructors should minimize the impact of high-stakes tests, while offering a diversity of formative assessment options in their courses. These methods could be particularly beneficial in male-stereotyped STEM fields where women are a minority in the classroom and may perceive the greatest risk of failure. Furthermore, while our emphasis is on differential performance as a function of gender, we anticipate similar phenomena may characterize the experiences of minority students, first-generation college students, and any other group more likely to suffer in high-stakes testing environments.

### The course deficit model

In conclusion, we return to our suggestion that certain features of the classroom environment can serve to erect barriers and create deficits in the performance of underserved students. According to the course deficit model, many barriers students face can be mitigated by instructional choices, such as the use of low-stakes, formative assessment techniques (described here), intentional use of role models as examples [33], and the removal of cues that foster stereotypes about the discipline [34]. Critically, the course deficit model, unlike the student deficit model, gives the instructor primary responsibility for designing his or her course to reduce barriers to success.

## Supporting information

S1 File Summary statistics. Tables A-C: statistical results for performance metrics and accounting for potential demographic predictors across nine introductory biology courses in fall 2016. We used AIC model-selection statistics to determine variables to include in the models. Significant variables are shown in bold. Tables D and E: descriptive statistics of normalized exam z-scores and percentage exam scores across nine introductory biology courses at the University of Minnesota, sorted by the proportion that exams account for in the final course grade. Table F: Statistical results for combined exam performance and final course grade across two semesters of BIOL 100, an introductory biology course for nonmajors students. Table G: Statistical results for combined exam performance and final course grade across two semesters of BIOL 300, an upper-division biology course for biology majors. Table H: Statistical results for combined exam performance and final course grade across two semesters of BIOL 202/203, a sequence of two lower-division biology courses for biology majors.
(DOCX)

S1 Table Individual results from fall 2016 (de-identified). Student data are combined (“Fa2016”) and sorted by courses as described in the manuscript.
(XLSX)
