# Supplementary material for: Can mixed assessment methods make biology classes more equitable?
Source: PLoS One. 2017 Dec 27;12(12):e0189610. doi: 10.1371/journal.pone.0189610 (PMC5744948; doi:10.1371/journal.pone.0189610)
Supplement: S1 File — Tables A-C: statistical results for performance metrics and accounting for potential demographic predictors across nine introductory biology courses in fall 2016. We used AIC model-selection statistics to determine variables to include in the models. Significant variables are shown in bold. Tables D and E: descriptive statistics of normalized exam z-scores and percentage exam scores across nine introductory biology courses at the University of Minnesota, sorted by the proportion that exams account for in the final course grade. Table F: Statistical results for combined exam performance and final course grade across two semesters of BIOL 100, an introductory biology course for nonmajors students. Table G: Statistical results for combined exam performance and final course grade across two semesters of BIOL 300, an upper-division biology course for biology majors. Table H: Statistical results for combined exam performance and final course grade across two semesters of BIOL 202/203, a sequence of two lower-division biology courses for biology majors. (DOCX) [file pone.0189610.s001.docx]

**Supplemental Tables A-C.** Statistical results for performance metrics and accounting for potential demographic predictors across nine introductory biology courses in fall 2016. We used AIC model-selection statistics to determine variables to include in the models. Significant variables are shown in bold.

| Table A. Mixed-effect regression analysis of predictors of *exam* performance in introductory biology courses. | | | | | |
| --- | --- | --- | --- | --- | --- |
| Parameter Estimates |  |  |  |  |  |
|  | **Estimate** | **Std. Error** | **df** | ***t*** | **Pr>*t*** |
| Intercept | -4.647 | 0.647 | 167.2 | -7.181 | <0.001 |
| Gender | 1.021 | 0.519 | 1067.5 | 1.966 | 0.050 |
| Age | 0.034 | 0.019 | 1068.4 | 1.746 | 0.081 |
| PercExam | 0.470 | 0.877 | 30.7 | 0.536 | 0.596 |
| Gender x PercExam | -2.205 | 1.085 | 1063.2 | -2.032 | 0.042 |
| ACT | 0.144 | 0.008 | 1045.2 | 18.919 | <0.001 |

| Table B. Mixed-effect regression analysis of predictors of performance on *non-exam* points in introductory biology courses. | | | | | |
| --- | --- | --- | --- | --- | --- |
| Parameter Estimates |  |  |  |  |  |
|  | **Estimate** | **Std. Error** | **df** | ***t*** | **Pr>*t*** |
| Intercept | 0.079 | 0.742 | 1071.0 | 0.106 | 0.915 |
| Gender | 1.028 | 0.599 | 1071.0 | 1.716 | 0.086 |
| Age | -0.055 | 0.022 | 1071.0 | -2.454 | 0.014 |
| PercExam | -0.299 | 0.997 | 1071.0 | -0.299 | 0.765 |
| Gender x PercExam | -1.574 | 1.252 | 1071.0 | -1.258 | 0.209 |
| ACT | 0.039 | 0.009 | 1071.0 | 4.421 | <0.001 |

| Table C. Mixed-effect regression analysis of predictors of *total course* performance in introductory biology courses. | | | | | |
| --- | --- | --- | --- | --- | --- |
| Parameter Estimates |  |  |  |  |  |
|  | **Estimate** | **Std. Error** | **df** | ***t*** | **Pr>*t*** |
| Intercept | -3.188 | 0.680 | 1071.0 | -4.686 | <0.001 |
| Gender | 1.541 | 0.549 | 1071.0 | 2.808 | 0.005 |
| Age | -0.009 | 0.020 | 1071.0 | -0.439 | 0.661 |
| PercExam | 0.569 | 0.914 | 1071.0 | 0.623 | 0.534 |
| Gender x PercExam | -2.983 | 1.147 | 1071.0 | -2.601 | 0.009 |
| ACT | 0.116 | 0.008 | 1071.0 | 14.445 | <0.001 |

**Supplemental Tables D-E.** Descriptive statistics of normalized exam z-scores and percentage exam scores across nine introductory biology courses at the University of Minnesota, sorted by the proportion that exams account for in the final course grade.

| Table D. Normalized exam z-scores across introductory biology courses. The value z represents the distance between the raw score and the class mean in units of the standard deviation. | | | | | | | | | | |
| --- | --- | --- | --- | --- | --- | --- | --- | --- | --- | --- |
| Course | Female Mean | Std. Deviation | N | Male Mean | Std. Deviation | N | Total mean | Std. Deviation | N | % Exams |
| 1 | **0.13** | 1.07 | 55 | 0.01 | 0.84 | 27 | 0.09 | 0.99 | 82 | 41 |
| 2 | **0.05** | 0.97 | 54 | 0.24 | 0.87 | 35 | 0.12 | 0.93 | 89 | 41 |
| 3 | **0.12** | 0.82 | 93 | 0.14 | 1.08 | 55 | 0.13 | 0.92 | 148 | 41 |
| 4 | **0.07** | 1.05 | 30 | 0.00 | 0.86 | 42 | 0.03 | 0.94 | 72 | 46 |
| 5 | **0.01** | 0.99 | 36 | 0.14 | 0.95 | 37 | 0.08 | 0.97 | 73 | 46 |
| 6 | **-0.12** | 0.93 | 78 | 0.31 | 0.86 | 82 | 0.10 | 0.92 | 160 | 50 |
| 7 | **0.01** | 1.04 | 87 | 0.03 | 0.76 | 28 | 0.02 | 0.98 | 115 | 52 |
| 8 | **-0.09** | 0.95 | 82 | 0.04 | 1.04 | 57 | -0.04 | 0.98 | 139 | 52 |
| 9 | **-0.17** | 0.97 | 122 | 0.28 | 1.00 | 77 | 0.01 | 1.01 | 199 | 52 |
| Total | **-0.02** | 0.97 | 637 | 0.16 | 0.94 | 440 | 0.05 | 0.96 | 1077 |  |

| Table E. Percentage exam scores across introductory biology courses. | | | | | | | | | | |
| --- | --- | --- | --- | --- | --- | --- | --- | --- | --- | --- |
| Course | Female Mean | Std. Deviation | N | Male Mean | Std. Deviation | N | Total mean | Std. Deviation | N | % Exams |
| 1 | 0.71 | 0.14 | 55 | 0.69 | 0.11 | 27 | 0.70 | 0.13 | 82 | 41 |
| 2 | 0.71 | 0.11 | 54 | 0.73 | 0.10 | 35 | 0.72 | 0.10 | 89 | 41 |
| 3 | 0.73 | 0.09 | 93 | 0.74 | 0.12 | 55 | 0.73 | 0.10 | 148 | 41 |
| 4 | 0.78 | 0.10 | 30 | 0.77 | 0.08 | 42 | 0.78 | 0.09 | 72 | 46 |
| 5 | 0.78 | 0.10 | 36 | 0.80 | 0.10 | 37 | 0.79 | 0.10 | 73 | 46 |
| 6 | 0.75 | 0.12 | 78 | 0.80 | 0.11 | 82 | 0.78 | 0.11 | 160 | 50 |
| 7 | 0.66 | 0.13 | 87 | 0.67 | 0.09 | 28 | 0.67 | 0.12 | 115 | 52 |
| 8 | 0.66 | 0.11 | 82 | 0.67 | 0.12 | 57 | 0.66 | 0.12 | 139 | 52 |
| 9 | 0.73 | 0.11 | 122 | 0.78 | 0.11 | 77 | 0.75 | 0.11 | 199 | 52 |
| Total | 0.72 | 0.12 | 637 | 0.75 | 0.12 | 440 | 0.73 | 0.12 | 1077 |  |

**Supplemental Tables F-G. Statistical Results for Three Case Studies.**

**Table F.** Statistical results for combined exam performance and final course grade across two semesters of BIOL 100, an introductory biology course for nonmajors students. *N* = 230

| Combined exam grade | |  | | | |
| --- | --- | --- | --- | --- | --- |
| Source | Type III Sum of Squares | df | Mean Square | F | Sig. |
| Corrected Model | **43.755** | **4** | **10.939** | **13.820** | **<0.001** |
| Intercept | **36.482** | **1** | **36.482** | **46.093** | **<0.001** |
| ACT | **37.543** | **1** | **37.543** | **47.434** | **<0.001** |
| Semester | 0.342 | 1 | 0.342 | 0.433 | 0.511 |
| SGender | 0.034 | 1 | 0.034 | 0.042 | 0.837 |
| Semester * SGender | **3.537** | **1** | **3.537** | **4.469** | **0.036** |

| Final course grade | |  | | | |
| --- | --- | --- | --- | --- | --- |
| Source | Type III Sum of Squares | df | Mean Square | F | Sig. |
| Corrected Model | **26.405** | **4** | **6.601** | **7.627** | **<0.001** |
| Intercept | **22.378** | **1** | **22.378** | **25.857** | **<0.001** |
| ACT | **23.092** | **1** | **23.092** | **26.682** | **<0.001** |
| Semester | 0.114 | 1 | 0.114 | 0.132 | 0.717 |
| SGender | 0.007 | 1 | 0.007 | 0.008 | 0.927 |
| Semester * SGender | 2.136 | 1 | 2.136 | 2.468 | 0.118 |

**Table G.** Statistical results for combined exam performance and final course grade across two semesters of BIOL 300, an upper-division biology course for biology majors. *N* = 164

| Combined exam grade | |  | | | |
| --- | --- | --- | --- | --- | --- |
| Source | Type III Sum of Squares | df | Mean Square | F | Sig. |
| Corrected Model | **68.586** | **4** | **17.146** | **29.191** | **<0.001** |
| Intercept | **60.008** | **1** | **60.008** | **102.158** | **<0.001** |
| ACT | **60.039** | **1** | **60.039** | **102.211** | **<0.001** |
| Semester | 1.625 | 1 | 1.625 | 2.766 | 0.098 |
| SGender | 2.784 | 1 | 2.784 | 4.739 | 0.031 |
| Semester * SGender | **2.763** | **1** | **2.763** | **4.704** | **0.032** |

| Final course grade | |  | | | |
| --- | --- | --- | --- | --- | --- |
| Source | Type III Sum of Squares | df | Mean Square | F | Sig. |
| Corrected Model | **66.558** | **4** | **16.639** | **27.736** | **<0.001** |
| Intercept | **57.724** | **1** | **57.724** | **96.220** | **<0.001** |
| ACT | **57.537** | **1** | **57.537** | **95.907** | **<0.001** |
| Semester | 1.303 | 1 | 1.303 | 2.172 | 0.142 |
| SGender | 0.898 | 1 | 0.898 | 1.496 | 0.223 |
| Semester * SGender | **5.224** | **1** | **5.224** | **8.708** | **0.004** |

**Table H.** Statistical results for combined exam performance and final course grade across two semesters of BIOL 202/203, a sequence of two lower-division biology courses for biology majors. *N* = 155 individuals over two semesters

| Combined exam grade | | | | |
| --- | --- | --- | --- | --- |
| Source | Numerator df | Denominator df | F | Sig. |
| Intercept | **1** | **155.114** | **26.397** | **<0.001** |
| ACT | **1** | **155.130** | **26.587** | **<0.001** |
| Semester | 1 | 153.477 | 0.005 | 0.945 |
| SGender | 1 | 155.008 | 1.009 | 0.317 |
| Semester * SGender | **1** | **153.477** | **4.365** | **0.038** |

| Final course grade | | | | |
| --- | --- | --- | --- | --- |
| Source | Numerator df | Denominator df | F | Sig. |
| Intercept | **1** | **153.639** | **20.698** | **<0.001** |
| ACT | **1** | **153.656** | **20.779** | **<0.001** |
| Semester | 1 | 152.276 | 0.009 | 0.925 |
| SGender | 1 | 153.706 | 0.176 | 0.675 |
| Semester * SGender | **1** | **152.276** | **6.321** | **0.013** |
